# Effects of rhodiola crenulata on mice hearts under severe sleep apnea

**DOI:** 10.1186/s12906-015-0698-0

**Published:** 2015-06-25

**Authors:** Mei-Chih Lai, Jaung-Geng Lin, Pei-Ying Pai, Mei-Hsin Lai, Yueh-Min Lin, Yu-Lan Yeh, Shiu-Min Cheng, Yi-fan Liu, Chih-Yang Huang, Shin-Da Lee

**Affiliations:** Graduate Institute of Chinese Medicine, China Medical University, Taichung, Taiwan; Graduate Institute of Clinical Medical Science, China Medical University and Hospital, Taichung, Taiwan; Department of Nursing, Master Program, Hungkuang University, Taichung, Taiwan; Department of Pathology, Changhua Christian Hospital, Changhua, Taiwan; Department of Medical Technology, Jen-The Junior College of Medicine, Nursing and Management, Miaoli, Taiwan; Department of Psychology, Asia University, Taichung, Taiwan; Department of Physical Therapy and Graduate Institute of Rehabilitation Science, China Medical University, No. 91, Hsueh-Shih Road, Taichung, Taiwan; Graduate Institute of Basic Medical Science, China Medical University and Hospital, Taichung, Taiwan; Department of Health and Nutrition Biotechnology, Asia University, Taichung, Taiwan; Department of Healthcare Administration, Asia University, Taichung, Taiwan

**Keywords:** Apoptosis, Caspase, Cardiac, Hypoxia, Rhodiola, Survival, VEGF

## Abstract

**Background:**

The goal of this study is to determine if Rhodiola Crenulata (RC) has protective effects on mice hearts with severe sleep apnea model.

**Methods:**

Sixty-four C57BL/6 J mice 5–6 months old were distributed into 4 groups i.e. Control group (21 % O_2_, 24 h per day, 8 weeks, n = 16); Hypoxia group (Hypoxia: 7 % O_2_ 60 s, 20 % O_2_ alternating 60 s, 8 h per day, 8 weeks, n = 16); Hypoxia + 90RC and Hypoxia + 270RC group (Hypoxia for 1st 4 weeks and hypoxia pretreated 90 mg/Kg and 270 mg/Kg Rhodiola Crenulata by oral gavage per day for 2nd 4 weeks, each n = 16). Excised hearts from 4 groups of mice were analyzed for heart weight index changes using H&E staining, TUNEL-positive assays and Western Blotting protein.

**Results:**

Cardiac widely dispersed TUNEL-positive apoptotic cells in mice hearts were less in Hypoxia + RC90 and Hypoxia + RC270 than those in Hypoxia. Compared with Hypoxia, the protein levels of Fas ligand, Fas death receptors, Fas-Associated Death Domain (FADD), activated caspase 8, and activated caspase 3 (Fas dependent apoptotic pathways) were decreased in Hypoxia + RC90, Hypoxia + RC270. The protein levels of Bad, Bax, t-Bid, activated caspase 9, activated caspase 3 (mitochondria dependent apoptotic pathway) were less in Hypoxia + RC90, Hypoxia + RC270 than those in hypoxia. The protein levels of Bcl2, Bcl-xL, p-Bad (Bcl2-realted anti-apoptotic pathway) and VEGF, p-PI3k, p-AKT (VEGF-related pro-survival pathway) were higher in Hypoxia + RC90, Hypoxia + RC270 than those in hypoxia.

**Conclusions:**

Our findings suggest that Rhodiola Crenulata have protective effects on chronic intermittent hypoxia-induced cardiac widely dispersed apoptosis via Fas-dependent and mitochondria-dependent apoptotic and VEGF-related pro-survival pathway.

## Introduction

Obstructive sleep apnea (OSA) is a sleep breathing disorder characterized by intermittent upper airway collapse during sleep and leads to sleep fragmentation [1]. Obstructive sleep apnea is often associated with cardiovascular diseases including coronary artery diseases and congestive heart failure [2, 3]. Noninvasive mechanical ventilation therapy reduced left ventricular end systolic and diastolic diameters, reduced the occurrence of cardiovascular complications in coronary heart disease with OSA [4]. Chronic intermittent hypoxia (CIH) showed various long-term cardiovascular pathophysiologic outcomes during sleep which are similar to OSA [1]. Chronic intermittent hypoxia was often used in animal models to simulate the experiment of obstructive sleep apnea [5, 6].

Rhodiola Crenulata, a species of Rhodiola, was often applied to avoid altitude sickness [7, 8] and it have been reported to decreased hypoxia-induced oxidative stress [8, 9]. In general, Rhodiola increased stress resistance, cardiopulmonary protection, and cardiovascular function [7]. Rhodiola rosea, a species of Rhodiola, as an adaptogen, have effects of anti-fatigue, anti-stress, anti-hypoxic, anti-cancer, and antioxidant in cell cultures, animals, or humans [10, 11]. Salidroside [2-(4-hydroxyphenyl)ethyl beta-D-glucopyranoside], a chemical component of Rhodiola Crenulata with phenylpropanoid glycoside was regarded as an active ingredient of Rhodiola [12]. In general, salidroside have been reported to protect cardiomyocytes against cardiotoxicity [13], stimulate mitochondrial biogenesis, protect endothelial dysfunction [14], enhance stem cells [15], induce erythropoietin [16]. In our previous study, salidroside showed protection through chronic intermittent hypoxia-induced Fas-dependent and mitochondria-dependent apoptotic pathways on mice hearts [5]. However, the effect of Rhodiola Crenulata on cardiovascular health is still unclear.

The apoptosis decreases cardiomyocytes in cardiomyopathies and shows also to be a predictor of cardiac diseases or heart failure [17]. From our previous studies, cardiac apoptosis were found in obesity [18, 19] smoking exposure [20], and under long-term but not short-term intermittent hypoxia [6, 21, 22]. The Fas receptor-dependent and mitochondria-dependent apoptotic pathways are the two major pathways triggering cardiac apoptosis [23]. The Fas receptor-dependent apoptotic (Type I) pathway is a key pathway triggering cardiac apoptosis [23]. The Fas binding the Fas receptor [23–25], trailed by Fas-receptor oligomerisation results in the death-inducing signal complex, begins with recruitment of the Fas-Associated Death Domain (FADD) adaptor protein [23]. The FADD activates caspase 8, cleaves pro-caspase 3 and undergoes autocatalysis forming active caspase 3, an effector caspase of apoptosis [24, 25].

The mitochondria-dependent (Type II) apoptotic pathway begins with the apoptosis-regulating protein Bcl-2 family, including anti-apoptotic Bcl-2 and Bcl-xL as well as pro-apoptotic Bad and Bax [23, 25, 26]. Pro-apoptotic Bcl2 family augments cytochrome *c* release from mitochondria [23, 25, 27]. Cytochrome *c* release into cytosol activates caspase-9, then caspase-3 completes the apoptotic program [23, 25]. Additionally, t-Bid was considered to be a key intracellular molecule signaling mediator from Fas to mitochondrial pathway since activated caspase 8 can cleave Bid to t-Bid then release cytochrome *c* to mitochondria-dependent apoptosis activity [23, 24]. In our previous studies, 8-week chronic intermittent hypoxia activated the Fas-dependent and mitochondria-dependent apoptotic pathways and increased cardiac apoptosis in rodent hearts [5, 6, 21, 22].

Vascular Endothelial Growth Factor (VEGF), a potent angiogenesis angiogenic growth factor, promotes survival, reduces Endothelial Cells apoptosis and activates PI3K/Akt signaling in human endothelial cells [28]. VEGF up-regulates pro-survival Akt/Bcl-2 signaling pathways as a survival factor for protecting cardiomyocyte against apoptosis [29]. Akt, as a key part in the phosphatidylinositide 3-kinase (PI3K) pathway, a vital spot regulatory kinase through pro-survival and protective effect in myocardium [30].

The effect of Rhodiola Crenulata on cardiac apoptosis in severe sleep apnea i.e. under chronic intermittent hypoxia is not understood. In the study, we investigate whether Rhodiola Crenulata have protective effects on mice heart with chronic intermittent hypoxia. We hypothesized that Rhodiola Crenulata may prevent cardiac apoptosis via Fas-dependent and mitochondrial-dependent anti-apoptotic pathways and VEGF-related pro-survival pathways in chronic intermittent hypoxia.

## Materials and Methods

### Animal model

The study was performed on sixty-four 5–6 months old C57BL/6 J mice were divided into four groups i.e. Control group (21 % O_2_, 24 h per day, 8 weeks, n = 16); Hypoxia group (Intermittent hypoxia: 7 % O_2_ 60 s and alternating 20 % O_2_ 60 s, 8 h per day, 8 weeks, n = 16); Hypoxia + 90RC and Hypoxia + 270RC group (Intermittent hypoxia for 1st 4 weeks; Intermittent hypoxia pretreated 90 mg/Kg and 270 mg/Kg Rhodiola Crenulata by oral gavage per day for 2nd 4 weeks, each n = 16). After exposure with normoxia or hypoxia and saline or RC, the hearts were excised and analyzed by heart weight index, H&E staining, TUNEL-positive assays and Western Blotting. Ambient temperature was kept at 25 °C and the animals were exposed to an artificial 12-h light–dark cycle, the light period which began at 7:00 am. Mice were provided with standard laboratory meals (Lab Diet 5001; PMI Nutrition International Inc., Brentwood, MO, USA) and water *ad libitum*. Protocols were proved by the Institutional Animal Care and Use Committee of China Medical University, Taichung, Taiwan.

### Rhodiola crenulata

Rhodiola Crenulata was provided by TCM Biotech International Corp (Taiwan). The extraction process was as follows: The raw material was analyzed and verified to be free of organic toxins and heavy metals (Pb, Hg, Cd, As, Cu) or below acceptable levels. The raw material was ground and sifted with a 20 mesh screen. The grainy powder was extracted and compressed then freeze dried to reduce water content to less than 8 %. The material was then ground to return to a powder form (water less 6 %). The powder was sifted then packaged in a sterile aluminum bag.

### Echocardiography

The trans-thoracic echocardiographic images of all mice were obtained using Philips M2424A ultrasound systems (Andover, MA) in anesthesia with 1 % isoflurane through a nose cone. The M-mode echocardiographic inspection was executed applying a 6–15 MHz linear transducer (15–6 L) through a parasternal long axis approach. The left ventricular M-mode dimensions on the stage of the papillary muscles contained inter-ventricular septum (IVS), left ventricular internal end-diastolic dimensions (LVIDd), left ventricular internal end-systolic dimensions (LVIDs), posterior wall thicknesses (LVPW), and fractional shortening (FS). FS% was calculated as a result of the equation FS% = [(LVIDd-LVIDs)/LVIDd]/100.

### Cardiac characteristics

The hearts of mice in the four groups were extracted and cleaned with PBS. The weights of the left ventricles and the whole heart were measured. Once weighting, the 6 hearts of mice in each group were soaked in formalin and added analyzed by Hematoxylin-eosin, Masson trichrome staining, DAPI staining and TUNEL assay. The other 6 hearts of mice in each group were cleaned, frozen, and added analyzed by Western Blotting. Besides, right tibia lengths were measured using a digital caliper and the left heart weight were weighed. The whole heart weight (WHW) to body weight (BW) ratio, the left ventricle weight (LVW) to body weight (BW) ratio, the left ventricle weight (LVW) to the to the whole heart weight (WHW) ratio, and the whole heart weight to tibia length and the left ventricle weight to tibia length ratio were determined for analysis.

### Tissue extraction

The left ventricle samples of cardiac tissue extracts were homogenized in a lysis buffer at a ratio of 100 mg tissue/1 ml buffer for 1 min to obtain cardiac tissue extracts. The homogenates were put on ice for 10 min then centrifuged at 12,000 *g* for 40 min twice. The supernatant was collected and stored at −70 °C.

### Electrophoresis and Western Blot

Protein concentration of heart tissue extracts was verified by the Bradford method (Bio-Rad Protein Assay, Hercules, CA, USA). Protein samples (50 μg/lane) were divided on a 10 % SDS polyacrylamide gel electrophoresis (SDS-PAGE) among a fixed voltage of 75 V. Electrophoresed proteins were relocated to polyvinylidene difluoride (PVDF) membrane (Millipore, Bedford, MA, 0.45 μm pore size) through a transfer equipment (Bio-rad). PVDF membranes were incubated in 5 % milk within TBS buffer. Primary antibodies containing Fas receptor, FADD, Bcl-xL, Bcl-2, Bax, t-Bid, caspase 8, caspase-9, caspase-3, p-Bad, VEGF, p-PI3k (Santa Cruz Biotechnology, Santa Cruz, CA, USA), Bad (BD Biosciences, San Jose, California, USA), p-Akt (Cell Signaling Technology, South San Francisco, CA, USA) and α-tubulin (Neo Markers, Fremont, CA, USA) were diluted to 1:500 into antibody binding buffer overnight on 4 °C. The immunoblots were cleaned in TBS buffer three times for 10 min then set in the 2nd antibody solution consisting of donkey anti goat IgG-HRP, goat anti-mouse IgG-HRP or goat anti-rabbit IgG-HRP (Santa Cruz) planned as one hour and diluted five hundred fold in a TBS buffer. The immunoblotted proteins were visualized applying an augmented chemiluminescence ECL western Blotting luminal Reagent (Santa Cruz, CA, USA), also quantified using a Fujifilm LAS-3000 chemiluminescence detection system (Tokyo, Japan).

### H&E staining, Masson trichrome staining, and TUNEL

The heart was extracted then soaked in formalin, then dehydrated via graded alcohols, after that embedded in paraffin wax. In heart samples, the 0.2-μm thick paraffin sections were cut from paraffin-embedded tissue blocks. The tissue samples were dewaxed using in xylene immersion, and then rehydrated. For Hematoxylin-eosin staining (H&E staining), the slices were dyed with hematoxylin and eosin. For Masson Trichrome Staining, the slices were dyed with Masson Trichrome. After lightly rinsing with water, the slides were dehydrated through graded alcohols then immersed in Xylene twice. Photomicrographs were obtained by Zeiss Axiophot microscopes. The Terminal Deoxynucleotide Transferase-mediated dUTP Nick End Labeling (TUNEL) assay were studied with an apoptosis detection kit (Roche Applied Science, Indianapolis, IN, USA) and observed that TUNEL-positive nuclei (fragmented DNA) fluoresced bright green at 450–500 nm. The mean number of TUNEL-positive cells were calculated for 5–6 individual fields x 2 slices x 3 regions of the left ventricle (the upper, the middle, the lower) removed from 6 mice hearts within all groups. All data counts were executed in two independent individuals within a blind study.

### Statistical analysis

The weight index, protein levels and percentage of TUNEL positive cells were compared with the Control, the Hypoxia, the Hypoxia + RC90 and the Hypoxia + RC270 groups by analysis of variance with pre-planned contrast comparison. In every case *P* < 0.05 was considered significant.

## Results

### Body weight and cardiac characteristics

Body weight, whole heart weight, left ventricular weight in the Hypoxia, the Hypoxia + RC90, and the Hypoxia + RC270 groups were similar to the Control group (Table 1). Whole heart weight (WHW), left ventricular weight (LVW), whole heart weight normalized by tibia length (WHW/Tibia), left ventricular weight normalized by tibia length (LVW/Tibia) in the Hypoxia + RC90, and the Hypoxia + RC270 groups were similar to the Control group. Inter-ventricular septum at diastole (IVSd), left ventricle posterior wall thicknesses (LVPW), left ventricle internal dimension at diastole (LVIDd), left ventricular internal end-systolic dimensions (LVIDs) in the Hypoxia, the Hypoxia + RC90, and the Hypoxia + RC270 groups were similar to the Control group (Table 1). Compared to the Control Group, fractional shortening (FS %) was decreased by chronic intermittent hypoxia about 6.2 %. Compared to the Hypoxia Group, the Fractional Shortening (FS %) was increased in the Hypoxia + RC90 group (4.8 %) and Hypoxia + RC270 group (8.0 %), respectively.

**Table 1:** Heart weight and echocardiographic indices

	Control	Hypoxia + Saline	Hypoxia + RC90	Hypoxia + RC270
Number of mice	8	8	8	8
Body weight (BW), g	21.56 ± 0.56	21.95 ± 1.32	21.79 ± 1.04	20.50 ± 1.44
Whole heart weight (WHW), g	0.108 ± 0.009	0.111 ± 0.007	0.109 ± 0.015	0.107 ± 0.008
Left ventricular weight (LVW), g	0.078 ± 0.009	0.077 ± 0.008	0.076 ± 0.015	0.078 ± 0.004
WHW/BW (x10^4^)	48.28 ± 5.62	50.74 ± 4.77	50.04 ± 8.22	52.73 ± 3.07
LVW/BW (x10^4^)	37.75 ± 4.43	32.65 ± 9.76	34.97 ± 8.26	38.83 ± 3.26
LVW/WHW	0.73 ± 0.08	0.64 ± 0.19	0.69 ± 0.06	0.74 ± 0.05
WHW/Tibia, g/mm (x10^4^)	58.74 ± 4.28	56.85 ± 16.99	60.79 ± 8.3	59.66 ± 4.72
LVW/Tibia, g/mm (x10^4^)	43.02 ± 4.95	39.60 ± 11.79	42.40 ± 8.48	43.81 ± 2.40
(IVSd), mm	0.75 ± 0.06	0.70 ± 0.04	0.72 ± 0.09	0.69 ± 0.04
(LVPWd), mm	0.77 ± 0.08	0.61 ± 0.09	0.64 ± 0.10	0.68 ± 0.09
(LVIDd), mm	3.74 ± 0.20	3.69 ± 0.30	3.94 ± 0.16	3.58 ± 0.31
(LVIDs), mm	2.45 ± 0.44	2.63 ± 0.33	2.62 ± 0.20	2.27 ± 0.32
Fractional Shortening (FS), %	34.80 ± 10.43	28.61 ± 7.05*	33.39 ± 4.29^#^	36.64 ± 5.47^##^

Values are Mean ± SD. *WHW/BW* whole heart weight normalized by body weight; *LVW/BW* left ventricular weight normalized by body weight; *LVW/WHW* left ventricular weight normalized by whole heart weight; *WHW/Tibia* whole heart weight normalized by tibia length; *LVW/Tibia* left ventricular weight normalized by tibia length; *IVSd* inter-ventricular septum at diastole; *LVPWd* left ventricular posterior wall thickness at diastole; *LVIDd* internal dimension at diastole of left ventricle; *LVIDs* internal dimension at systole of left ventricle; *FS*: fractional shortening (LVIDd-LVIDs/LVIDd x100)

* P < 0.05, significant differences from the Control group. ^#^P < 0.05, ^##^P < 0.01, significant differences from the Hypoxia group

### Cardiac histopathological changes of left ventricle

**Fig. 1:**
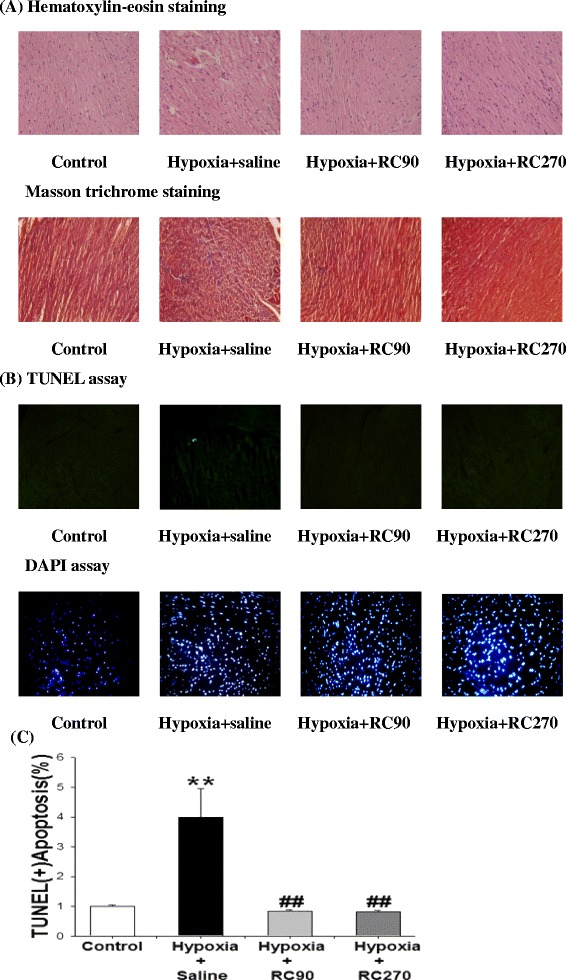
**a** Representative histopathological analysis of cardiac sections from left ventricles in the Control, the Hypoxia, the Hypoxia + RC90, and the Hypoxia + RC270 RC groups was performed with Hematoxylin and eosin (H&E) staining and Masson trichrome staining method. The images were magnified by 400 times. **b** The Representative stained apoptotic cells of cardiac sections from left ventricles in the Control, the Hypoxia, the Hypoxia + RC90, and the Hypoxia + RC270 RC groups were measured by TUNEL assay with dark background (upper panels, green spots) and staining with DAPI (lower panels, blue spots). The images were magnified by 400 times. **c** Bars presents the percentage of TUNEL positive cells relative to the total DAPI cells (n = 6 in each group). **P < 0.01, denotes significant differences from the Control group. ^##^P < 0.01 denotes significant differences from the Hypoxia group

To determine if there were changes in cardiac architecture, a histopathological analysis of ventricular tissue stained with hematoxylin and eosin was performed. We viewed 400X magnified images and found that the ventricular myocardium in the Control group showed normal architecture and interstitial space. We found abnormal myocardial architecture and the increased interstitial space in the Hypoxia group. These myocardial architecture abnormalities in the Hypoxia + RC90, Hypoxia + RC270 groups were less than those in the Hypoxia group (Fig. 1a).

### TUNEL-positive apoptotic cells of left ventricle

To analyze the apoptotic activity in cardiac tissues, the apoptotic cells and total cells were measured by TUNEL assay and DAPI staining respectively. Hypoxia groups stained with TUNEL assay had the most TUNEL-positive cardiac cells when compared to the Control group, Hypoxia + RC90 or the Hypoxia + RC270 groups. Decreases in the number of TUNEL-positive cardiac cells were detected in the Hypoxia + RC90 and the Hypoxia + RC270 groups compared with the Hypoxia group. TUNEL-positive cells relative to DAPI-stained nuclei cells in the hypoxia group about 4.1 % is higher than those in the Hypoxia + RC90 and the Hypoxia + RC270 group both about 0.9 % (Fig. 1).

### Upstream components of cardiac Fas receptor dependent apoptotic pathways

**Fig. 2:**
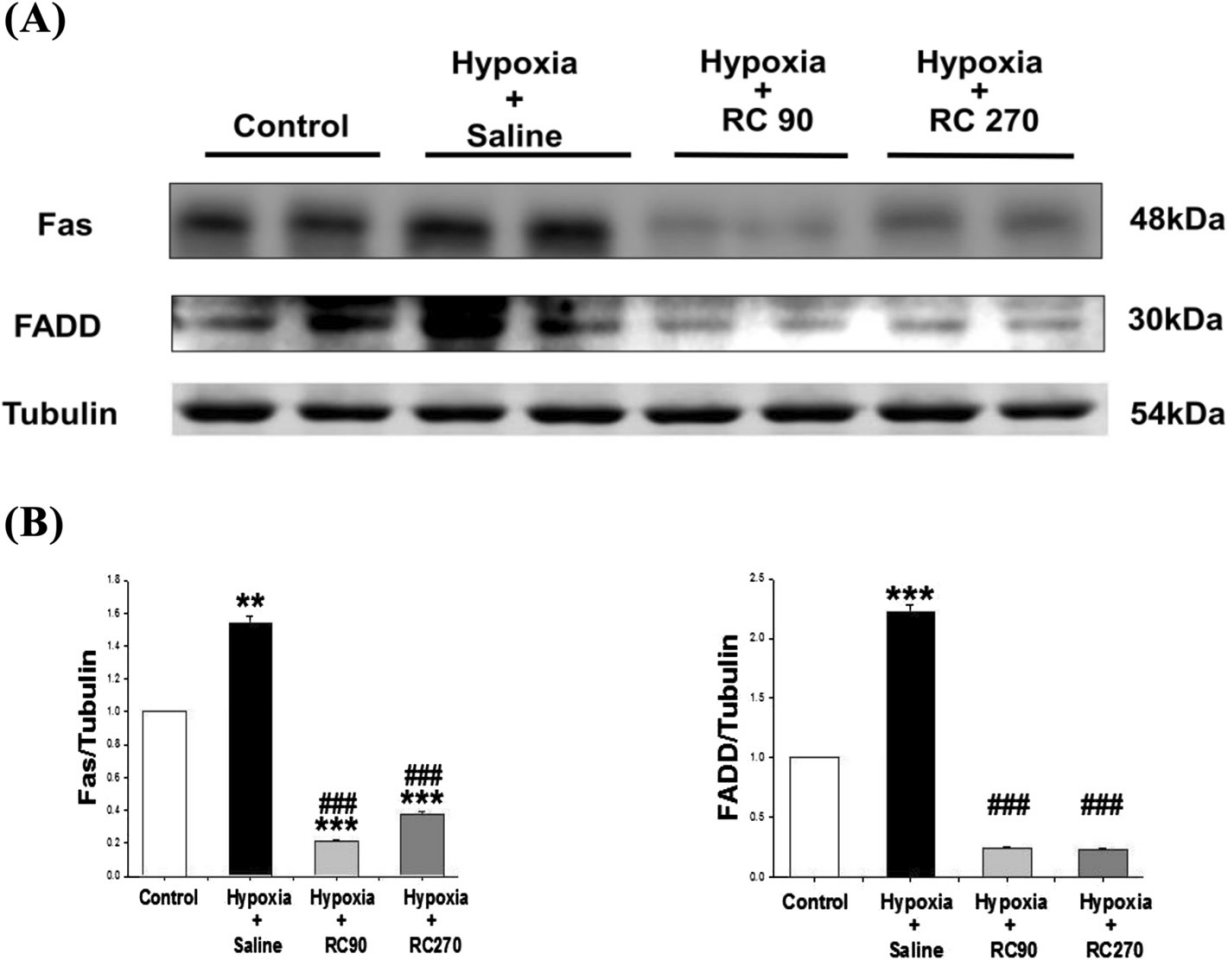
**a** The representative protein products of Fas receptor and Fas-associated death domain (FADD) extracted from the left ventricles of excised hearts in the Control, the Hypoxia, the Hypoxia + RC90, and the Hypoxia + RC270 RC groups were measured by Western Blot analysis. **b** The bars represent the relative protein quantification of Fas receptor and Fas-associated death domain (FADD) on the basis of α-tubulin, respectively, and indicates Mean values ± SD (n = 6 in each group). **P < 0.01, ***P < 0.001, are the significant differences from the Control. ^###^P < 0.001, are the significant differences from the Hypoxia group

To determine the upstream components of cardiac Fas receptor dependent apoptotic signaling pathways in mice with treatment of RC under chronic intermittent hypoxia, protein levels of Fas receptor and FADD in the excised hearts of the Control, the Hypoxia, the Hypoxia + RC90 and the Hypoxia + RC270 groups were examined by Western blotting. Compared with the Control group, Fas receptor and FADD were significantly increased in the Hypoxia group (Fig. 2). Fas receptor and FADD in the Hypoxia + RC90 and Hypoxia + RC270 groups were significantly lower than those in the Hypoxia group (Fig. 2).

### Upstream components of cardiac mitochondrial-dependent apoptotic pathways

**Fig 3:**
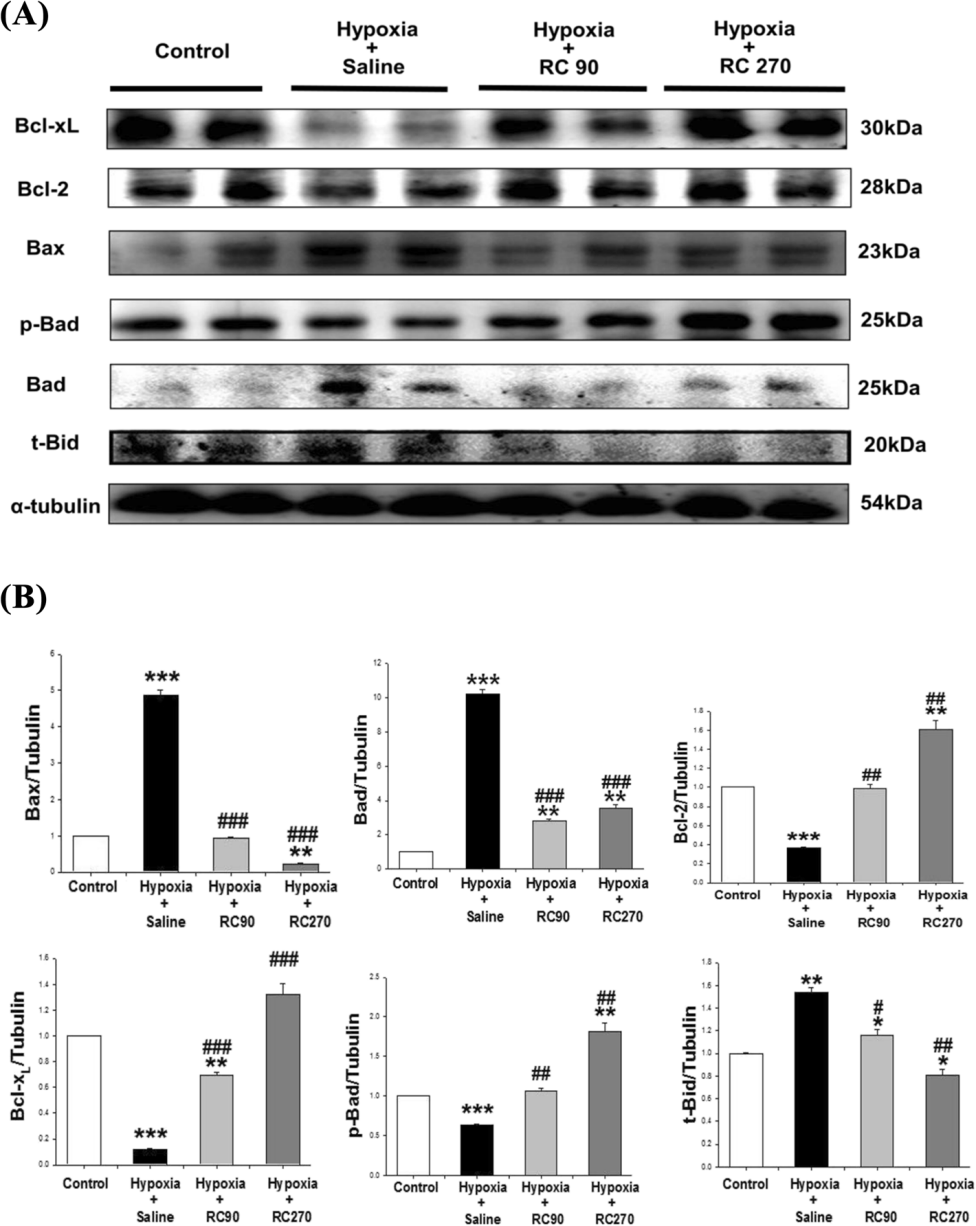
**a** The representative protein products of Bcl-xL, Bcl2, Bax, p-Bad, Bad and t-Bid extracted from the left ventricles of excised hearts in the Control, the Hypoxia, the Hypoxia + RC90, and the Hypoxia + RC270 RC groups were measured by Western Blotting analysis. **b** The bars represent the relative protein quantification of Bcl-xL, Bcl2, Bax, p-Bad, Bad and t-Bid on the basis of α-tubulin and indicates Mean values ± SD (n = 6 in each group). *P < 0.05, **P < 0.01, ***P < 0.001, are the, significant differences from the Control. ^#^P < 0.05, ^##^P < 0.01, ^###^P < 0.001are the significant differences from the Hypoxia group

To evaluate the cardiac Bcl-2 family in mitochondria-dependent apoptotic pathways in mice with chronic intermittent hypoxia and treatment with RC, we analyzed the protein levels of the Bcl-2 family (Bcl-xL, Bcl-2, Bax, Bad, p-Bad) in the excised hearts of the Control group, the Hypoxia, the Hypoxia + RC90 and Hypoxia + RC270 groups by Western Blotting. Mitochondrial related anti-apoptotic protein of Bcl-xL, Bcl-2, p-Bad in the Hypoxia + RC90 and Hypoxia + RC270 groups were higher than those in the Hypoxia group. Mitochondrial related pro-apoptotic proteins of Bax and Bad were much higher in the Hypoxia group than the Control group as well as the Hypoxia + RC90 and Hypoxia + RC270 groups (Fig. 3). The protein level of pro-apoptotic t-Bid, a main intracellular molecule signaling mediator from Fas to mitochondrial pathway, in the Hypoxia + RC90 and Hypoxia + RC270 groups were significantly lower than the protein in the Hypoxia group (Fig. 3).

### Downstream components of cardiac Fas and mitochondrial dependent apoptosis

**Fig. 4:**
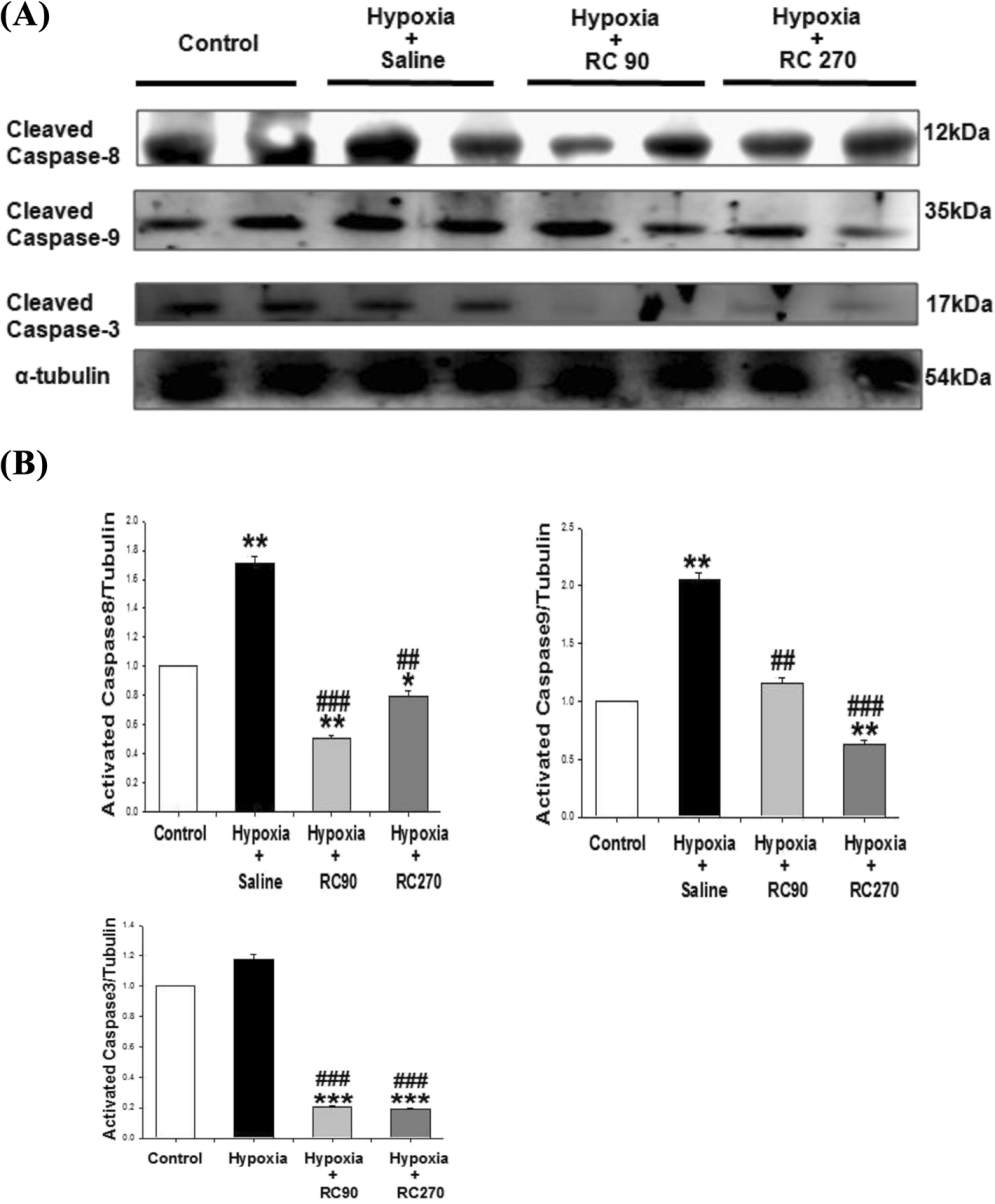
**a** The representative protein products of activated caspase 8, activated caspase 9, and activated caspase 3 extracted from the left ventricles of excised hearts in the Control, the Hypoxia, the Hypoxia + RC90, and the Hypoxia + RC270 RC groups were measured by Western Blotting analysis. **b** The bars represent the relative protein quantification of activated caspase 8, activated caspase 9, and activated caspase 3 on the basis of α-tubulin, respectively, and indicates Mean values ± SD (n = 6 in each group). **P < 0.01, ***P < 0.001 are the significant differences from the Control. ^##^P < 0.01, ^###^P < 0.001, are the significant differences from the Hypoxia group

To realize the downstream components of cardiac Fas receptor (caspase 8 and 3) and mitochondrial (caspase 9 and 3) dependent apoptotic pathways, the caspase 8, 9 and 3 were measured by Western blotting in hearts excised from the four experimental groups. Western blot analysis showed that the protein products of activated caspase 8, 9, and 3 were higher in the Hypoxia groups than the Control group. The protein level of activated caspase 8, 9, and 3 in the Hypoxia + RC90 and Hypoxia + RC270 groups were much lower than in the Hypoxia group (Fig. 4).

### Cardiac VEGF-related pro-survival pathway

**Fig. 5:**
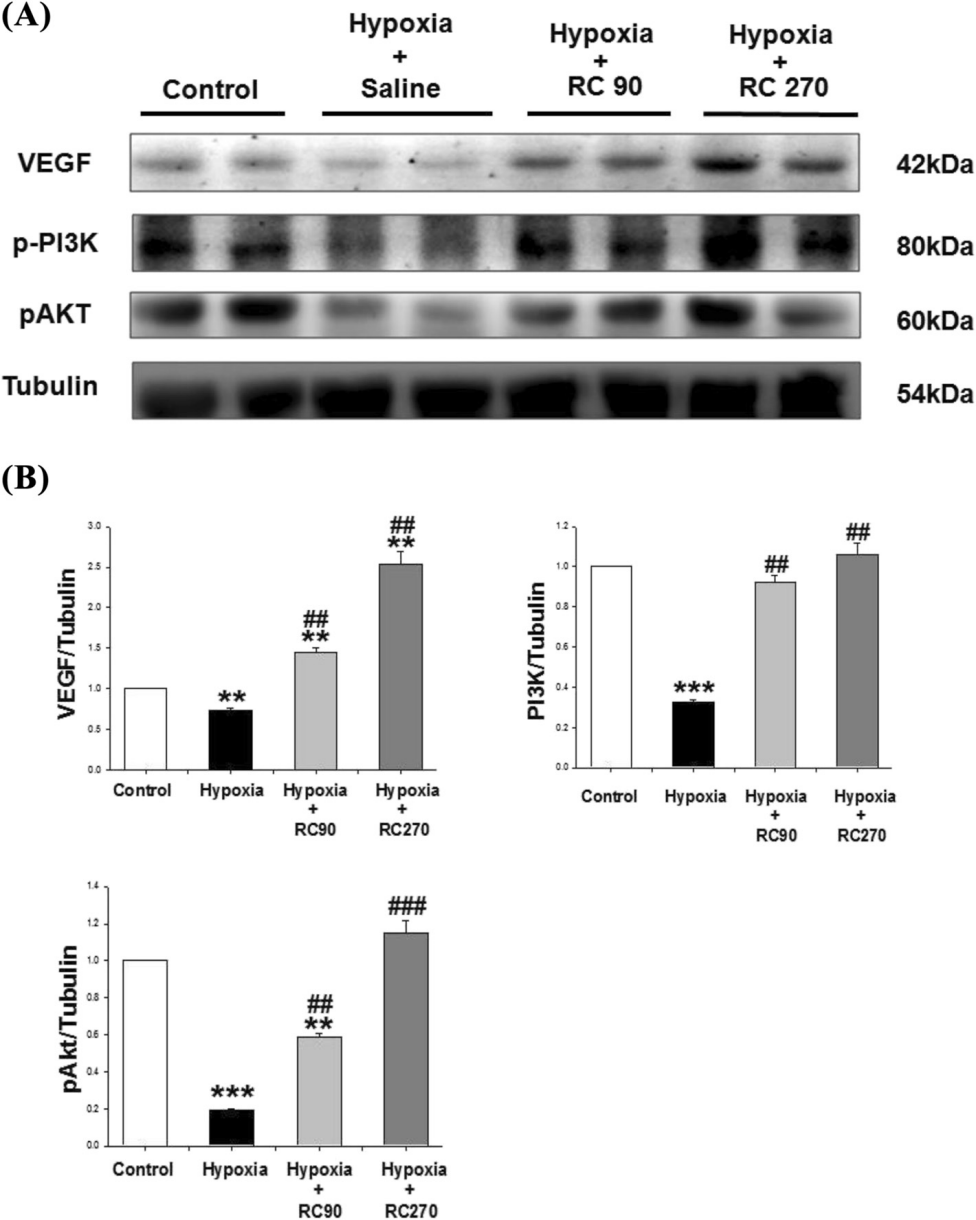
**a** The representative protein products of activated enhance VEGF-related pro-survival pathway (VEGF, p-PI3k, p-AKT) cardiac pro-survival pathway extracted from the left ventricles of excised hearts in the Control, the Hypoxia, the Hypoxia + RC90, and the Hypoxia + RC270 RC groups were measured by Western Blotting analysis. **b** The bars represent the relative protein quantification of VEGF, p-PI3k, and p-AKT on the basis of α-tubulin, respectively, and indicates Mean values ± SD (n = 6 in each group). **P < 0.01, ***P < 0.001 are the significant differences from the Control. ^##^P < 0.01, ^###^P < 0.001, are the significant differences from the Hypoxia group

To understand the VEGF-related pro-survival pathway in mice with chronic intermittent hypoxia and treatment with RC, the VEGF, p-PI3k, p-Akt were measured by Western blotting in hearts excised from the four experimental groups. Western blot analysis showed that the protein products of VEGF, p-PI3k and p-Akt were higher in the RC groups than the Control group (Fig. 5). The protein levels of VEGF, p-PI3k and p-Akt in the Hypoxia + RC90 and Hypoxia + RC270 groups were much higher than those in the Hypoxia group (Fig. 5).

## Discussion

### Major findings

**Fig. 6:**
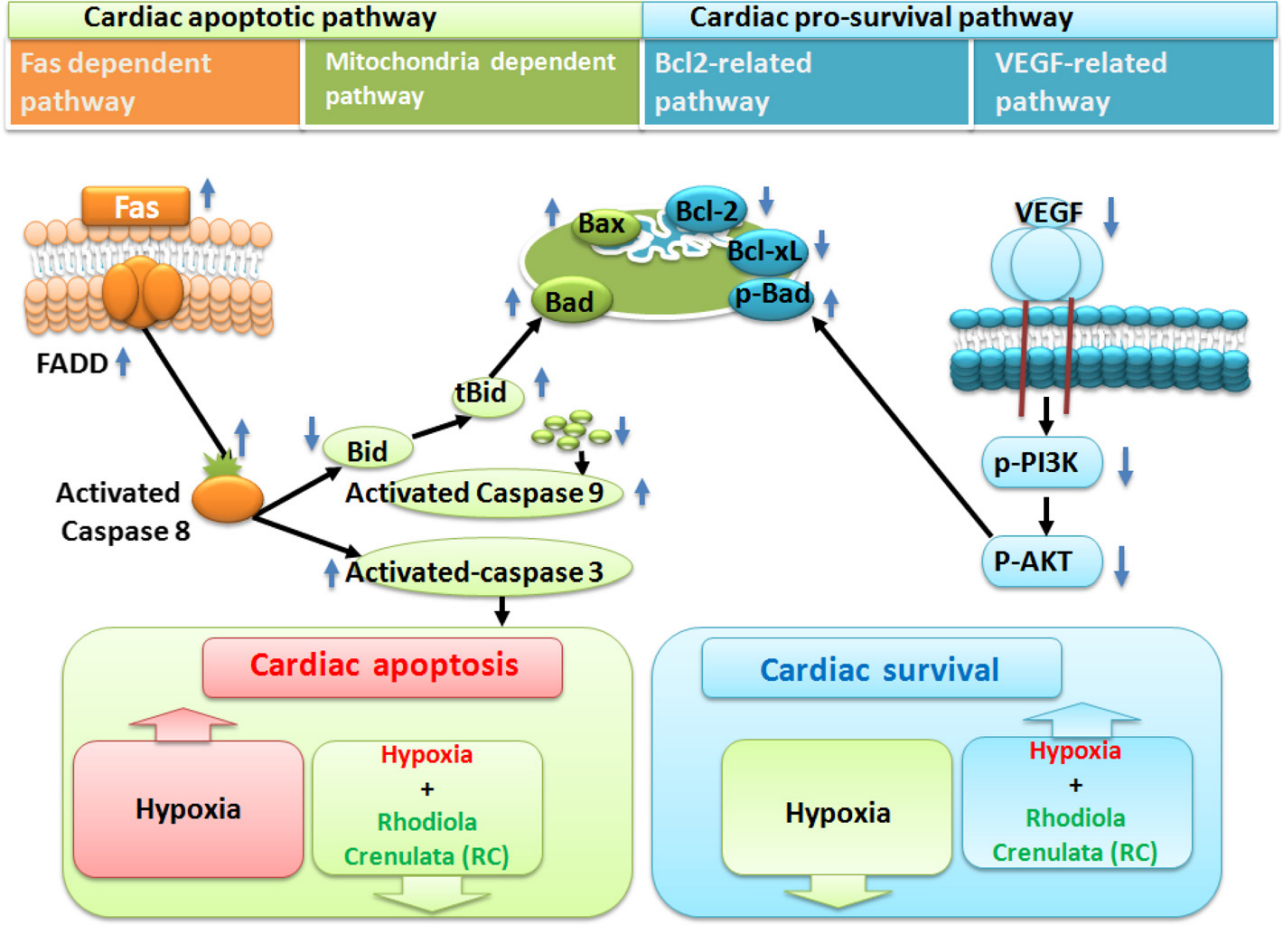
Our hypothesis that Rhodiola Crenulata suppressed chronic intermittent hypoxia-induced cardiac Fas-dependent, mitochondria-dependent apoptotic pathways and enhanced chronic intermittent hypoxia-induced cardiac VEGF-related pro-survival pathway. The data is based on chronic intermittent hypoxia increases in pro-apoptotic Fas, FADD, activated caspase 8, activated caspase 3 (left pathway, Fas-dependent apoptotic pathway), and chronic intermittent hypoxia decreases anti-apoptotic Bcl-xL, Bcl-2, and p-Bad, increases t-Bid, Bad, Bax, activated caspase-9, and activated caspase-3 (middle pathway, mitochondria-dependent apoptotic pathway), and chronic intermittent hypoxia increased pro-survival protein VEGF, p-PI3k and p-AKT (right pathway, pro-survival VEGF-related pathway). Rhodiola Crenulata decreases chronic intermittent hypoxia-induced cardiac Fas-dependent and mitochondrial-dependent apoptotic pathways, but increases chronic intermittent hypoxia-induced cardiac VEGF-related pro-survival pathway. The upward arrows and downward arrows represent the respective increases and decreases

The summarized conclusions are (1) Rhodiola Crenulata treatment improved chronic intermittent hypoxia-decreased cardiac fractional shortening. (2) The activity of the cardiac Fas receptor-dependent apoptotic pathway in mice with chronic intermittent hypoxia was significantly decreased with treatment of Rhodiola Crenulata, which data is based on decreases in Fas, FADD, activated caspase 8, activated caspase 3 compared to the Hypoxia group. (3) The activity of the cardiac mitochondrial-dependent apoptotic pathway in mice with chronic intermittent hypoxia was significantly decreased with treatment of Rhodiola Crenulata, which is based on increases in anti-apoptotic protein levels, Bcl-xL, Bcl-2 and p-Bad, as well as decreases in pro-apoptotic protein levels, Bad, Bax, t-Bid, activated caspase-9 and activated caspase 3. (4) The activity of the cardiac VEGF-related pro-survival pathway in mice with chronic intermittent hypoxia was significantly increased with treatment of Rhodiola Crenulata, which is based on increased in pro-survival protein VEGF, p-PI3k and p-AKT level compared to the Hypoxia group. After integrating our findings with previous apoptotic theories, we propose a hypothesized diagram in Fig. 6, which shows that Rhodiola Crenulata treatment prevents chronic intermittent hypoxia-induced cardiac Fas-dependent apoptotic pathway (Fas, FADD, activated caspase 8, activated caspase 3), prevents chronic intermittent hypoxia-induced cardiac mitochondrial-dependent apoptotic pathway (t-Bid, Bad, Bax, activated caspase-9, and activated caspase-3 as well as decreased anti-apoptotic Bcl-xL, Bcl-2, p-Bad), and enhance chronic intermittent hypoxia-induced cardiac VEGF-related pro-survival pathway (VEGF, p-PI3k and p-AKT) (Fig. 6).

### Experimental design

In humans, Rhodiola Crenulata extract treatment of 800 mg daily for 7 days before ascent and 2 days during mountaineering of Hehuan Mountain in Taiwan prevented the headaches of Acute Mountain Sickness [31]. The dosage of Rhodiola Crenulata powder 100 mg/Kg and 500 mg/Kg, once daily for 4 weeks, ameliorate sucrose-induced acute hyperglycemia by gavage in mice [32]. Our current study of treatment with Rhodiola Crenulata in mice was designed as 90 mg/Kg and 270 mg/Kg by oral gavage. Our study suggests that the current dosage did protect chronic intermittent hypoxia-induced cardiac widely dispersed apoptosis. The reasons why long-term intermittent hypoxia was designed as 8 weeks were based on our previous studies. Long-term intermittent hypoxia planned for 8 weeks could cause abnormal myocardial architecture and increased pro-apoptotic BNIP3 and Bad proteins on rat’s hearts [21] as well as could induce cardiac widely dispersed apoptosis [5, 22]. Therefore, our study design of 8 weeks chronic intermittent hypoxia as negative control group and Rhodiola Crenulata treated for 4 weeks was validated based on the current findings. Besides, we should caution that any Rhodiola Crenulata-induced anti-apoptotic effect in the present investigation might be caused by various factors and cannot be isolated to any specific mechanism, such as effects of salidroside, effects of tyrosol, anti-oxidation, inhibiting free radicals, enhancing stem cells, inducing erythropientin or other unclear factors.

Rhodiola Crenulata appeared to prevent chronic intermittent hypoxia-induced abnormal myocardial architecture and cardiac dysfunction. We suggest that Rhodiola Crenulata treatment may prevent chronic intermittent hypoxia-induced abnormal myocardial architecture and progression. Rhodiola (not Rhodiola Crenulata) increases myocardial performance resulting in increased cardiac output in STZ-induced diabetic rats [33].

Cardiac widely dispersed apoptosis TUNEL-positive cells showed attenuation after treatment with Rhodiola Crenulata. This paper shows the therapeutic benefit of Rhodiola Crenulata treatment on chronic intermittent hypoxia-induced cardiac apoptosis. Salidroside, active ingredients of Rhodiola Crenulata, protected the cardiomyocytes against cardiotoxicity [13]. Salidroside had protective effects on cardiomyocytes necrosis [34]. In our previous study, Salidroside protected chronic intermittent hypoxia-induced cardiac apoptosis on mice hearts [5].

In our study, Rhodiola Crenulata treatment was shown significantly to prevent chronic intermittent hypoxia-induced cardiac Fas receptor-dependent apoptosis as determined by decreases in hypoxia-induced cardiac Fas receptors, FADD, activated caspase-8 levels and activated caspase-3 levels in mice. Our study suggests that Rhodiola Crenulata treatment will prevent chronic intermittent hypoxia-enhanced Fas-dependent and mitochondrial-dependent apoptotic pathway. In our previous study, salidroside treatment decreased chronic intermittent hypoxia-induced cardiac Fas ligand, Fas death receptors, FADD, activated caspase 8, and activated caspase 3 in mice hearts [5].

Our study suggests that Rhodiola Crenulata treatment prevented mitochondrial-dependent apoptotic pathway in mice with chronic intermittent hypoxia as discovered with increases anti-apoptotic Bcl2 family members (Bcl-xL, Bcl-2, p-Bad) and decreases in Bax, Bad, activated caspase-9 levels and activated caspase-3 within chronic intermittent hypoxia-induced cardiac apoptosis. Our study suggests that Rhodiola Crenulata treatment will prevent chronic intermittent hypoxia-enhanced the intracellular signaling mediator Bid from the Fas pathway to the mitochondrial pathway. Salidroside, protected the cardiomyocytes against doxorubicin-induced cardiotoxicity by limiting unnecessary oxidative stress and activating a Bcl2-mediated survival signaling pathway, protecting cardiac dysfunction, and decreasing cardiomyocyte apoptosis [13].

Our study suggests that the protein levels of VEGF-related pro-survival pathway (VEGF, p-PI3k, p-AKT) were decreased after chronic intermittent hypoxia on mice hearts as well as Rhodiola Crenulata prevented chronic intermittent hypoxia-induced cardiac widely dispersed apoptosis. Salidroside was found to drive effective antioxidant properties and protect cells from apoptosis by the PI3K/Akt pathway [35]. In our previous study, salidroside had cardiac protection through chronic intermittent hypoxia-induced apoptosis [5]. In two studies, salidroside protected against hypoxia-induced cardiomyocytes necrosis and apoptosis by increasing VEGF levels [36], as well as increased Akt phosphorylation, cardiomyocytes against injury by PI3K/Akt pathway and increased PI3K antioxidant enzyme [37]. Salidroside and Tyrosol, two isolated compounds of Rhodiola, prevented apoptosis in H9c2 cells, reduced caspase-3 activity, reduced cytochrome c release and showed the anti-apoptotic effect of the combination of the two components was more beneficial than that of salidroside and tyrosol independently [38].

Radix et Rhizoma Rhodiolae Kirilowii, a species of Rhodiola, enhances myocardial angiogenesis by increasing VEGF in rats with hypoxia [39]. Rhodiola Crenulata at 90 mg/Kg and 270 mg/Kg have protective effects on chronic intermittent hypoxia-induced cardiac widely dispersed apoptosis via Fas-dependent and mitochondria-dependent apoptotic pathway and VEGF-related pro-survival pathway. In summary, our study suggests that Rhodiola Crenulata treatment in mice with chronic intermittent hypoxia prevents Fas-dependent and mitochondrial-dependent apoptotic pathways and enhances VEGF-related cardiac pro-survival pathway.

### Hypothesized clinical application

This study showed that Rhodiola Crenulata treatment protects from cardiac dysfunction, damage and widely dispersed apoptosis under chronic intermittent hypoxia, an animal model of severe sleep apnea. Chronic intermittent hypoxia is regarded as one of the major components of obstructive sleep apnea [40]. Since cardiac tissues are not easily extracted from human hearts, the preventive effects of Rhodiola Crenulata on cardiac widely dispersed apoptosis in the animal study might provide new therapeutic information to prevent cardiac apoptosis in patients with severe Obstructive Sleep Apnea or hypoxia. Since Rhodiola Crenulata treatment could prevent cardiac damage after chronic intermittent hypoxia, we might further hypothesized that Rhodiola Crenulata might be applied to patients with severe sleep apnea to prevent cardiac apoptosis or heart failure and increase cardiac survival. Further clinical studies are required to clarify our clinical hypotheses.
